# Evaluating the 8‐Year Association Between Cognition and Beta Amyloid in the Dallas Lifespan Brain Study Cohort

**DOI:** 10.1002/brb3.71684

**Published:** 2026-09-15

**Authors:** Joshua David, Isabella Torres, Evan Smith, Ekarin Pongpipat, Denise Park

**Affiliations:** ^1^ School of Behavioral and Brain Sciences The University of Texas at Dallas Richardson Texas USA; ^2^ Psychology Department Bradley University Peoria Illinois USA; ^3^ Center For Vital Longevity Dallas Texas USA

**Keywords:** Alzheimer's disease, amyloid, Amyloid Cascade Hypothesis, cognition

## Abstract

**Background:**

Mounting evidence suggests beta amyloid (Aβ) is not the sole initiating driver of cognitive decline in Alzheimer's disease (AD). This study explores whether there is an association between cognitive decline over eight years and Aβ.

**Methods:**

Data from 48 participants in the Dallas Lifespan Brain Study (DLBS) were analyzed in the present study. Prior to selection, participants were classified as Aβ^+^ or Aβ^−^ based on global Aβ deposition. All 24 Aβ^+^ participants with complete data for the purposes of the present study were sex‐ and age‐matched with an Aβ^−^ participant and assorted into two respective Aβ groups. Cognitive decline across episodic memory, working memory, processing speed, and reasoning was then compared between the two groups over an 8‐year observation period.

**Results:**

An association between cognitive decline over 8 years and the Aβ group was observed in reasoning but not across episodic memory, working memory, and processing speed. Still, there were differences between the two groups that reached significance on an episodic memory task (*p* = 0.035) and trended toward significance on an encompassing episodic memory composite (*p* = 0.057). Post‐hoc comparisons revealed these differences were only significant at epoch 1. Broad declines in cognition were also found over the observation period, irrespective of Aβ group.

**Conclusion:**

Our results contribute to the mounting evidence that suggests Aβ is not the sole initiating driver of cognitive decline in AD. However, the present study has limitations, including a relatively small sample size that must be accounted for when considering the null findings to prevent overinterpretation. Future work should validate our results in larger, similarly controlled samples and model additional AD biomarkers and risk factors to explore different mechanisms for cognitive decline in AD.

## Graphical abstract

The present study investigated the 8‐year association between beta amyloid (Aβ) and cognitive decline to weigh in on the validity of the amyloid cascade hypothesis, which posits that Aβ drives the downstream processes leading to cognitive decline in Alzheimer's disease (AD). While Aβ was associated with decline in reasoning, the effect was clinically weak, and Aβ was otherwise not associated with episodic memory, working memory, or processing speed, altogether suggesting that Aβ may not be the sole initiating driver of cognitive decline in AD.

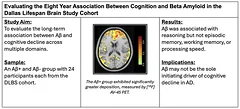

## 1 Introduction

Extracellular beta‐amyloid (Aβ) deposition has been considered a hallmark of Alzheimer's disease (AD) for some time (Hardy and Allsop 1991). While the exact pattern of deposition within the brain varies, recent advances in neuroimaging have revealed that the process begins prior to clinically meaningful cognitive impairment. Regardless, some studies have observed subtle decrements to cognition in association with Aβ during this preclinical stage of AD (Farrell et al. 2018; Petersen et al. 2016; Lim et al. 2013; Storandt et al. 2009). Previous histopathological and genetic studies have revealed similar implications, together supporting a model wherein Aβ initiates a cascade of events that eventually leads to cognitive impairment (Karran et al. 2011). This model was termed the amyloid cascade hypothesis.

Mounting evidence, however, suggests that Aβ cannot fully explain the cognitive deficits associated with AD. A systematic review by Parent et al. (2023) determined that only seven of the 17 evaluated studies reported a significant association between Aβ accumulation and cognitive decline. Furthermore, Hanseeuw et al. (2023) evaluated a plurality of sequential models of cognitive decline over 10 years and found an Aβ‐independent pathway to cognitive impairment that was initiated by entorhinal cortex tau aggregation and mediated by low hippocampal volume. As opposed to these recent studies, research conducted approximately more than a decade ago found broad associations between Aβ and cognitive decline across multiple abilities (Petersen et al. 2016; Lim et al. 2013; Storandt et al. 2009). These differences may be a result of the inability of prior studies to control for relevant cognitive risk factors, whether due to a lack of data collected regarding them or an already limited group of participants to compose a sample from. Thus, analyses on controlled samples remain necessary to exclusively evaluate Aβ‐initiated cognitive decline.

Another pertinent consideration in studies of aging pathologies such as Aβ is the ubiquitous challenge of longitudinal data collection, given that health concerns and mortality affect long‐term subject retention. An implication of this challenge is that many past longitudinal studies have been constrained to a relatively narrow band of time (Burke et al. 2019). This is a salient limitation, as Jagust and Landau (2021) estimated that it takes six years to transition from Aβ negativity to positivity and a subsequent 14 years to present clinically meaningful cognitive impairment. This finding emphasizes the necessity for longer longitudinal studies to accurately characterize the relationship between Aβ and cognitive decline.

Given the sparsity of controlled, long‐term longitudinal studies on Aβ and cognitive decline, the present study will focus on a cohort of cognitively unimpaired individuals over an eight‐year observation period. We selected 48 participants from the completed Dallas Lifespan Brain Study (DLBS), split into 24 Aβ^+^ and 24 sex‐ and age‐matched Aβ^−^ participants. In summary, the purpose of this study is to evaluate the eight‐year association between Aβ and cognitive decline in a controlled sample. We hypothesize that the Aβ^+^ group will exhibit greater cognitive decline than the Aβ^−^ group across the analyzed domains during this period.

## 2 Methods

### 2.1 Participants

Forty‐eight cognitively unimpaired participants within the range of 50–87 years old were selected from the DLBS. Each participant underwent an [^18^F] AV‐45 (^18^F‐florbetapir) PET at epoch 1 and completed a cognitive battery at epoch 1, epoch 2, and epoch 3 (M_1‐2_ = 3.73 years, SD_1‐2_ = 0.40 years; M_2‐3_ = 4.54 years, SD_2‐3_ = 0.85 years). The participant selection procedure was conducted in the subsequently described manner. First, 24 Aβ^+^ participants were selected based on availability of neuroimaging and cognitive data, as well as a Global Standardized Uptake Value ratio (SUVr) greater than 1.09 (composite regions are listed in *PET Preprocessing)* at baseline. For each Aβ^+^ participant, a same‐sex participant with a similar age (±5 years), complete data, and a Global SUVr less than 1.05 was placed in the Aβ^−^ group. The aforementioned Aβ^+^ threshold was validated by Farrell et al. (2021), and the Aβ^−^ threshold was implemented in consideration of the decision to dichotomize the biologically continuous process of Aβ deposition. Refer to Table 1 for sample characteristics.

**TABLE 1:** Characteristics for the entire sample and both groups are listed. The sample and both group columns are subdivided into three additional columns for the rows with domain composites and episodic memory tasks. The epoch each column represents is in order of the variable subscripts such that the left column represents epoch 1, the middle represents epoch 2, and the right represents epoch 3. The cognitive measures tabulated are z‐scored. The non‐parametric Geriatric Depression Scale Score (GDSS) variable is represented by the median score and interquartile range while the remaining numerical variables are represented by their mean and standard deviation. Two‐sided independent samples *t*‐tests and a Mann–Whitney *U* Test (for GDSS) were performed on select numerical variables to investigate their differences between the two groups. Some demographic variables were not compared due to the matching procedure, and cognitive data was not compared to prevent redundancy with primary analyses. (†) One participant did not possess data for the CIR task while another did not complete a blood pressure measurement.

	All Participants			Aβ^−^ Group			Aβ^+^ Group			*p*‐value
**N**	48			24			24			—
**Age**	70.1 (9.3)			69.9 (9.4)			70.4 (9.4)			—
**Female (%)**	75			75			75			—
**Years of Education**	15.6 (1.9)			15.9 (2.2)			15.3 (1.6)			0.367
**Systolic Blood Pressure_1_ (mmHg)**	133.0 (19.9)			128.3 (18.2)			137.8 (20.8)†			0.102
**Diastolic Blood Pressure_1_ (mmHg)**	81.4 (10.1)			79.9 (10.1)			82.9 (10.1)†			0.315
**Geriatric Depression Scale Score_1_**	1.0 (4.0)			1.0 (3.3)			1.5 (4.0)			0.567
**MMSE_1_**	28.5 (1.2)			28.8 (1.0)			28.3 (1.3)			0.183
**Global SUVr_1_**	1.10 (0.16)			1.01 (0.04)			1.20 (0.17)			< 0.001
**Episodic Memory_1|2|3_**	0.00 (0.88)	−0.09 (0.81)	−0.63 (1.04)	0.30 (0.93)	0.00 (0.79)	−0.40 (0.96)	−0.30 (0.72)	−0.17 (0.84)	−0.85 (1.09)	—
**Working Memory_1|2|3_**	0.00 (0.75)	−0.02 (0.77)	−0.29 (0.70)	−0.02 (0.62)	−0.06 (0.81)	−0.19 (0.72)	0.02 (0.87)	0.03 (0.74)	−0.39 (0.68)	—
**Processing Speed_1|2|3_**	0.00 (0.86)	−0.46 (0.96)	−0.44 (1.16)	−0.06 (0.89)	−0.50 (0.89)	−0.35 (1.12)	0.06 (0.85)	−0.43 (1.02)	−0.53 (1.22)	—
**Reasoning_1|2|3_**	0.00 (0.84)	−0.09 (0.86)	−0.41 (0.94)	0.03 (0.93)	−0.20 (0.97)	−0.29 (0.87)	−0.03 (0.76)	0.02 (0.74)	−0.54 (1.00)	—
**HIR_1|2|3_**	0.00 (1.00)	−0.05 (0.83)	−0.50 (1.00)	0.26 (1.10)	0.08 (0.85)	−0.31 (0.95)	−0.26 (0.83)	−0.17 (0.80)	−0.69 (1.03)	—
**CIR_1|2|3_**	0.00 (1.00)	0.00 (1.00)	0.00 (1.00)	0.14 (1.08)	0.10 (1.02)	0.31 (0.92)	−0.15 (0.91)†	−0.10 (0.99)	−0.31 (1.00)	—
**HDR_1|2|3_**	0.00 (1.00)	−0.15 (0.91)	−0.66 (1.00)	0.32 (1.00)	−0.01 (0.88)	−0.40 (0.98)	−0.32 (0.91)	−0.28 (0.95)	−0.91 (0.99)	—
**HRC_1|2|3_**	0.00 (1.00)	−0.06 (0.98)	−0.73 (1.41)	0.34 (0.99)	−0.06 (0.94)	−0.50 (1.23)	−0.34 (0.91)	−0.06 (1.04)	−0.95 (1.56)	—

Abbreviations: CIR = CANTAB Verbal Memory Immediate Recall, HDR = Hopkins Delayed Recall, HIR = Hopkins Immediate Recall, HRC = Hopkins Recognition Correct.

Additional relevant exclusion criteria from the DLBS itself included the following: a score less than 26 on the Mini‐Mental State Examination (MMSE) at epoch 1 and less than 22 at the subsequent epochs, loss of consciousness for more than 10 min, present alcoholism, history of psychosis, major neurological or psychiatric disorders, heart attacks or heart surgeries, left‐handedness, and cancers treated systematically with radiation or chemotherapy within the prior 5 years. The lowered MMSE cutoff score at the subsequent epochs was implemented to allow for age‐related decline and prevent attrition, though the lowest observed score across the entire DLBS was 24. The study was conducted in accordance with the University of Texas Southwestern Medical Center (UTSW) and the University of Texas at Dallas Institutional Review Boards. All participants provided written informed consent and were debriefed according to human investigations committee guidelines.

### 2.2 Cognitive Tasks and Domain Composites

We evaluated performance on certain episodic memory, working memory, processing speed, and reasoning tasks conducted in the DLBS, representing a diverse range of cognitive domains. Scores on the tasks were first z‐scored using the sample mean and standard deviation at baseline and subsequently averaged by cognitive domain to create four domain composite scores. Due to the recorded vulnerability of episodic memory to AD, we additionally chose to evaluate performance on the four constituent tasks used to construct the episodic memory composite. These tasks were Hopkins Immediate Recall (HIR), Hopkins Delayed Recall (HDR), Hopkins Recognition Correct (HRC), and CANTAB Verbal Memory Immediate Recall (CIR). The constituent tasks for the processing speed composite were Digit Comparison and WAIS‐III Digit Symbol, for the working memory composite were CANTAB Spatial Working Memory and the WAIS‐III Letter Number Sequencing Task, and for the reasoning composite were Raven's Matrices and ETS Letter Sets. DLBS procedures for the aforementioned tasks can be found in Park et al. (2025). Both the tasks and their procedures remained consistent across the three epochs.

### 2.3 MRI Acquisition

Participants were scanned using a 3T Philips Achieva (Philips, Best, the Netherlands) scanner at UTSW's Advanced Imaging Research Center with an eight‐channel head coil at baseline and the two follow‐up evaluations. High‐resolution anatomic images were collected with a T1‐weighted magnetization‐prepared rapid gradient‐echo sequence (MP‐RAGE) with 160 sagittal slices, a field of view of 204 × 256 × 150 mm, a voxel size of 1 × 1 × 1 mm^3^, a repetition time of 8.1 ms, an echo time of 3.7 ms, and a flip angle of 12°.

### 2.4 PET Acquisition

Patients were injected with a 370‐MBq (10 mCi) bolus of [^18^F] AV‐45 (^18^F‐florbetapir; Avid Radiopharmaceutical/Eli Lilly) 30 min prior to scanning in a Siemens ECAT HR PET scanner (Siemens, Munich, Germany) at UT Southwestern. A two‐minute scout was acquired to ensure full brain coverage and that there was no planar rotation. Fifty minutes after injection, PET data was collected using a dynamic emission acquisition, including two 5‐min frames. Immediately after, an internal rod source transmission scan was acquired for 7 min. The transmission image was reconstructed using back‐projection and a 6 mm full width at half‐maximum Gaussian filter with four iterations, 16 subsets, and a 3 mm full width at half‐maximum ramp filter. Refer to Park et al. (2025) for more information.

### 2.5 MRI and PET Preprocessing

First, each participant's T1‐weighted anatomical MP‐RAGE scan was processed using FreeSurfer 5.3 (Martinos Center for Biomedical Imaging, MA, USA). These parcellated images were manually edited by a well‐trained team if necessary, and quality was verified by a separate research team. Next, the PET data was motion‐corrected to the first frame using the FSL flirt command with 12 degrees of freedom, a mutual information cost function, and trilinear interpolation. After motion correction, the frames were averaged to create a mean PET image. The mean PET image was then co‐registered to the participant's T1w image at the corresponding timepoint with the FSL flirt command with the same motion correction parameters. The Desikan‐Killiany atlas was then used to create eight bilateral regions of interest (ROIs) for each subject: the anterior and posterior cingulate, as well as the lateral prefrontal, orbitofrontal, precuneus, lateral parietal, lateral occipital, and lateral temporal regions. These regions were specifically chosen due to past analysis of the DLBS cohort noting them as particularly vulnerable to Aβ deposition (Rodrigue et al. 2012). Tracer counts were separately extracted for each of these regions, and SUVr values were computed using the whole cerebellum as the reference region. These eight SUVr values were averaged to create a global SUVr composite reflecting widespread Aβ burden across the cerebral cortex, excluding the motor and sensory cortices.

### 2.6 Statistical Analyses

In the first set of analyses, four two‐way repeated measures ANOVAs were performed using domain composite scores as the dependent variable with Aβ group and epoch as independent variables. Next, an additional four two‐way repeated measures ANOVAs were performed using episodic memory task scores as the dependent variable with Aβ group and epoch as independent variables. Last, post‐hoc Bonferroni‐adjusted pairwise comparisons were conducted on significant or near‐significant associations using estimated marginal means. Due to our participant matching procedure, we did not control for the effects of sex or age in our models to maintain power. These analyses were performed on SPSS v29.0.0.0, and line graphs were created with R v4.5.2.

**FIGURE 1:**
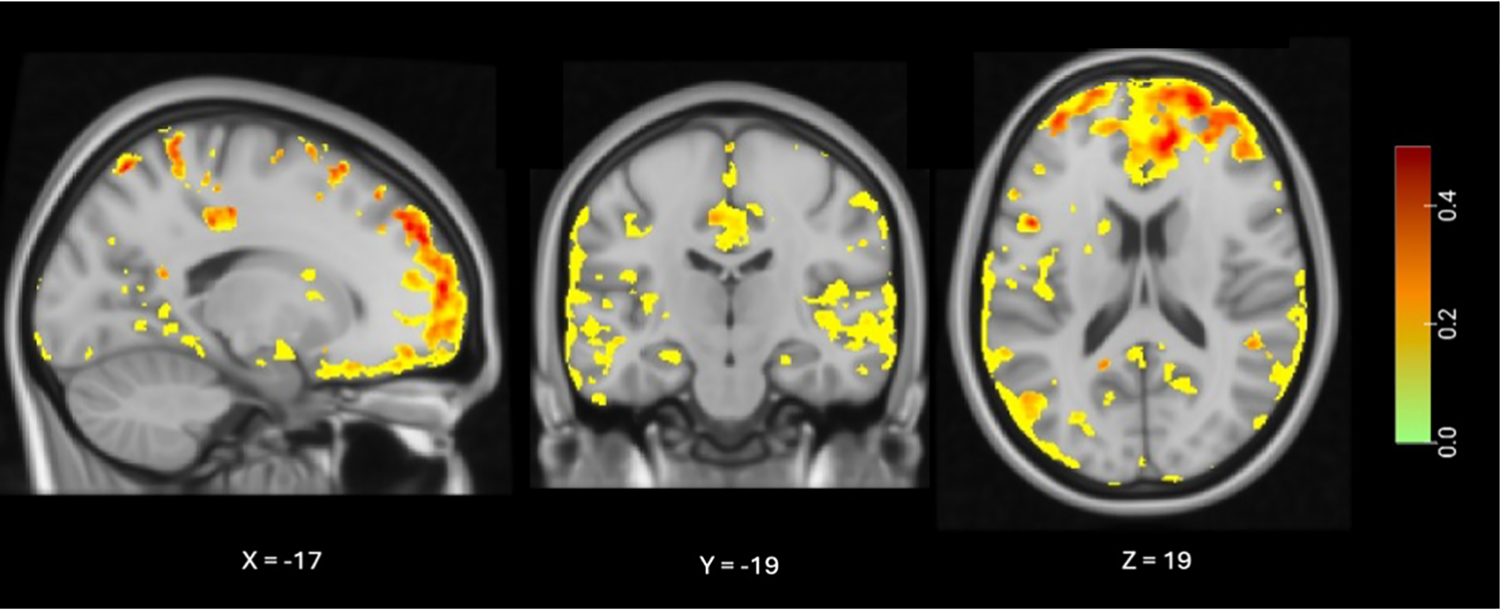
[^18^F]AV‐45 SUVr difference map displaying the difference in Aβ deposition between the two groups (Aβ^+^ group—Aβ^−^ group). The greatest differences were in the prefrontal and orbitofrontal cortices, though sparse, significant voxels are present across many regions.

To visualize spatial differences in Aβ deposition between the two groups, a difference map was also rendered (Figure 1). First, T1‐weighted images were warped to MNI space. These warps were subsequently applied to the SUVr images using ANTs. To create the difference map, *fsl glm* was used to run a voxel‐wise independent samples *t*‐test, corrected to achieve an overall *α* = 0.05 (*p* = 0.001, NN = 3, bi‐sided, 18 voxels) using ANFI's 3dFWHMx and 3dClustSim. Positive and negative clusters were formed separately, and significant clusters were formed if at least 18 contiguous voxels (touching via face, edge, corner, or a combination of the three) had a *p*‐value < 0.001.

## 3 Results

### 3.1 Group Comparison of Aβ Deposition

An independent samples *t*‐test was performed to examine group differences in Aβ deposition (see *Group Comparison of Aβ Deposition* for the complete procedure). After cluster correction, the Aβ^+^ group showed significantly more Αβ deposition in all eight ROIs used to compute the Global SUVr metric (see Figure 1 and Table 2).

**TABLE 2:** Significant between‐group differences in Aβ deposition within the eight ROIs used to compute the Global SUVr metric. Volume overlap represents the total number of voxels that overlap between the significant voxels from Figure 1 and each ROI. Percent overlap represents the volume overlap divided by the volume of the respective ROI. The max intensity coordinates are in MNI space.

ROI	Volume overlap (mm^3^)	% Overlap	Max intensity			
			*t*‐statistic	x	y	z
**Anterior Cingulate**	6378	69.30	5.54	−2	29	23
**Posterior Cingulate**	5294	42.53	6.58	6	−15	41
**Orbitofrontal**	8182	34.02	5.99	−18	44	−19
**Precuneus**	4199	25.52	5.95	9	−61	45
**Lateral Prefrontal**	36773	41.60	7.35	47	42	−17
**Lateral Parietal**	16974	33.69	6.81	62	−52	33
**Lateral Occipital**	2514	18.51	7.61	−44	−85	−12
**Lateral Temporal**	16300	42.80	6.97	−55	3	−34

### 3.2 Expected Findings in Accordance With the Hypothesis

We hypothesized that the Aβ^+^ group would exhibit greater cognitive decline than the Aβ^−^ group across the analyzed domains during the observation period. In accordance with this, we expected to see a significant effect of the Aβ group × epoch interaction term on domain composite and episodic memory task scores. For further clarity, a significant effect of the Aβ group would reflect differences in scores but not differences in decline, while a significant effect of epoch would reflect longitudinal decline irrespective of the Aβ group.

### 3.3 Longitudinal Changes in Domain Composite Scores

**FIGURE 2:**
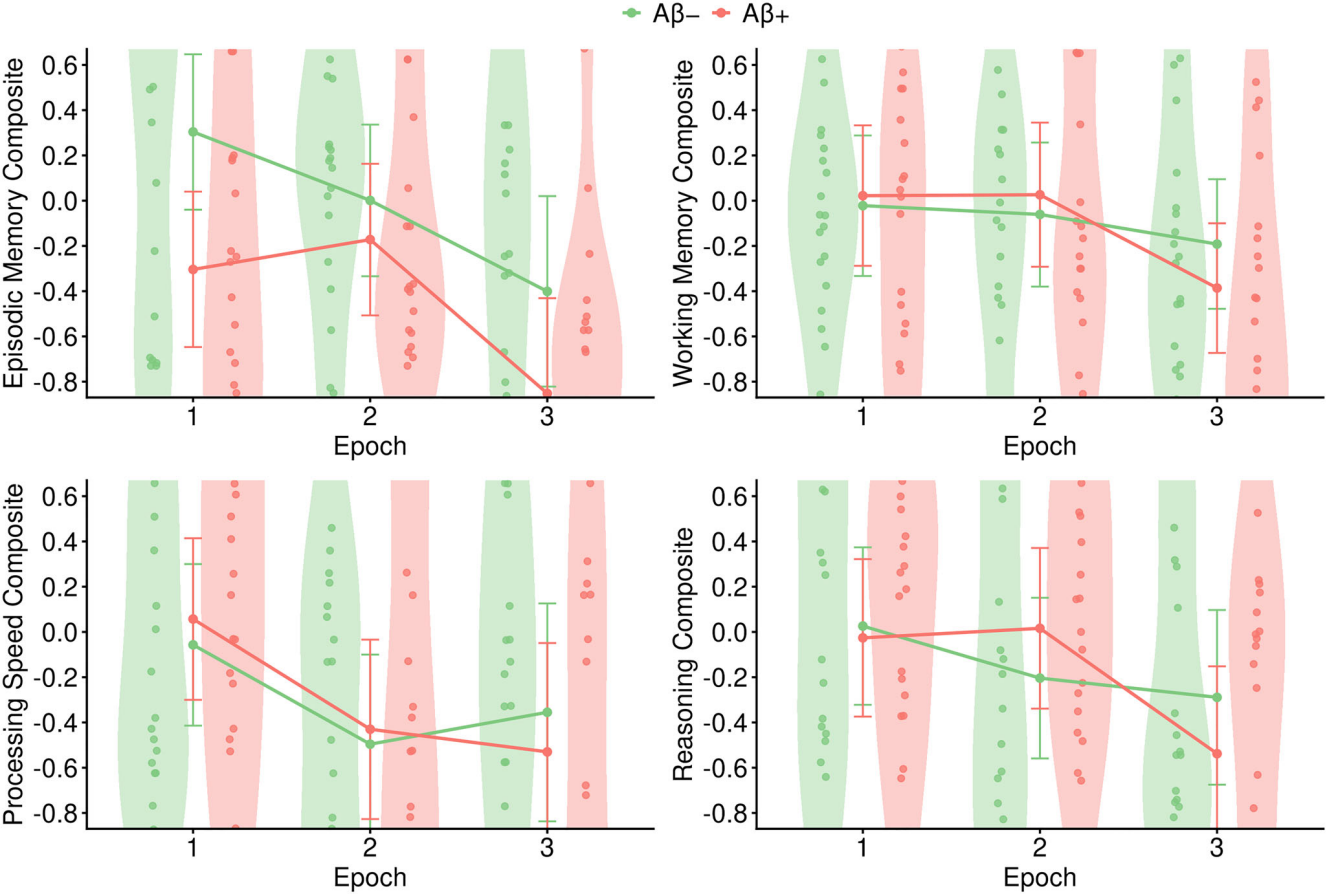
Line graphs of domain composite scores over time, separated by group. 95% CIs are included at each point. Group‐specific violin plots have also been overlayed to present more granular detail of the score distribution at each epoch. The orange and green lines represent the Aβ^+^ and Aβ^−^ groups, respectively.

Figure 2 presents the trajectory of domain composite scores over time. Both groups exhibited decline from epoch 1 to epoch 3 on each composite. Specifically, the Aβ^−^ group exhibited declines of 0.70 z‐units on the episodic memory composite, 0.32 z‐units on the reasoning composite, 0.30 z‐units on the processing speed composite, and 0.17 z‐units on the working memory composite, while the Aβ^+^ group exhibited declines of 0.55 z‐units, 0.51 z‐units, 0.59 z‐units, and 0.41 z‐units on those tasks, respectively. Despite this, the 95% CIs overlapped at all points and thus did not provide evidence for significant differences in domain composite scores between the two groups.

### 3.4 Analysis of Domain Composite Scores

Table 3 presents the results from the four two‐way repeated measures ANOVAs using domain composite scores as the dependent variable and Aβ group and epoch as independent variables. There was a significant effect of epoch on the four domain composite scores, reflecting longitudinal decline irrespective of Aβ group. A near‐significant effect of the Aβ group was observed on the episodic memory composite score (*F* = 3.80, *η* ^2^ = 0.08, *p* = 0.057). A significant Aβ group x epoch interaction was found with the reasoning composite score (*F* = 4.42, *η* ^2^ = .09, *p* = 0.015), indicating that Aβ differentially contributes to changes in reasoning over time. Post‐hoc analyses revealed a significant difference in episodic memory composite score between the two groups at epoch 1 (*p* = 0.015) but no significant differences between the two groups on the reasoning composite.

**TABLE 3:** Results from the four two‐way repeated measures ANOVAs using domain composite scores as the dependent variable and group and epoch as independent variables.

	Episodic memory			Working memory			Processing speed			Reasoning		
Effect	*F*	𝞰^2^	*p*	*F*	𝞰^2^	*p*	*F*	𝞰^2^	*p*	*F*	𝞰^2^	*p*
**Epoch**	13.62	0.23	< 0.001	7.07	0.13	0.001	14.17	0.24	< 0.001	14.94	0.25	< 0.001
**Group**	3.80	0.08	0.057	0.01	0.00	0.913	0.00	0.00	0.994	0.01	0.00	0.910
**E × G**	1.43	0.03	0.245	1.55	0.03	0.217	1.24	0.03	0.294	4.42	0.09	0.015

E × G represents the interaction term between epoch and group.

### 3.5 Longitudinal Changes in Episodic Memory Task Scores

**FIGURE 3:**
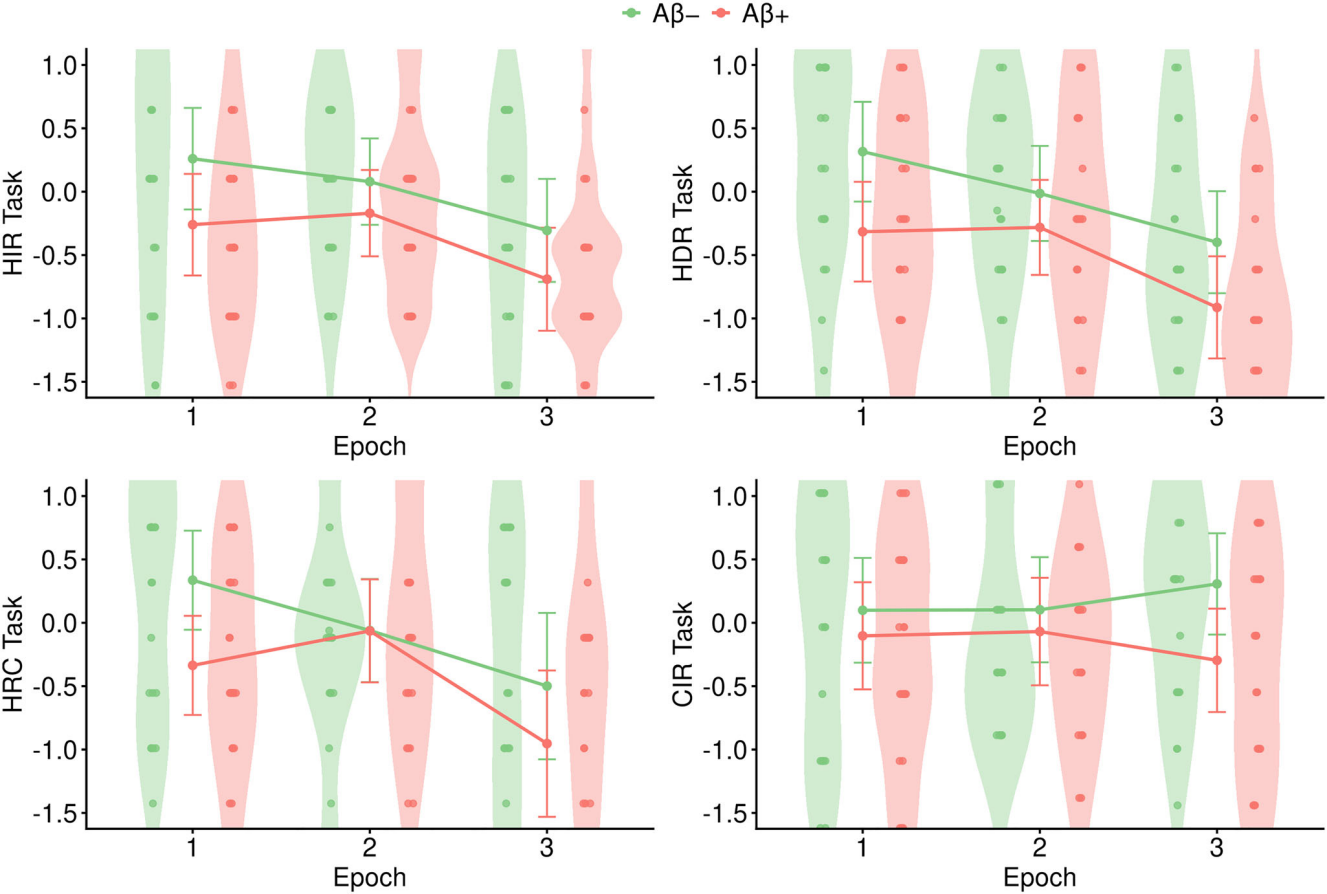
Line graphs of episodic memory task scores over time, separated by group. 95% CIs are included at each point. Group‐specific violin plots have also been overlayed to present more granular detail of the score distribution at each epoch. The orange and green lines represent the Aβ^+^ and Aβ^−^ groups, respectively. Abbreviations: CIR = CANTAB Verbal Memory Immediate Recall, HIR = Hopkins Immediate Recall, HDR = Hopkins Delayed Recall, HRC = Hopkins Recognition Correct.

Figure 3 presents the change in episodic memory task scores over time. With the sole exception of the Aβ^−^ group on the CIR task, both groups exhibited decline from epoch 1 to epoch 3 on each task. Specifically, the Aβ^−^ group had score declines of 0.84 z‐units on the HRC task, 0.72 z‐units on the HDR task, and 0.61 z‐units on the HIR task, while the Aβ^+^ group exhibited declines of 0.61 z‐units, 0.59 z‐units, and 0.43 z‐units, respectively, as well as a 0.20 z‐unit decline on the CIR task. Despite this, the 95% CIs overlapped at all points, and thus, did not provide evidence for any differences in episodic memory task scores between the two groups.

### 3.6 Analysis of Episodic Memory Task Scores

Table 4 presents the results from the four two‐way repeated measures ANOVAs using episodic memory task scores as the dependent variable and group and epoch as independent variables. With the exception of the CIR task, there was a significant effect of epoch on all task scores, reflecting longitudinal decline irrespective of Aβ group. There was a significant difference between the two groups on the HDR task (*F* = 4.74, *η* ^2^ = 0.09, *p* = 0.035) but no significant group x epoch interaction terms on any task scores. Similar to the episodic memory composite, post‐hoc analyses revealed a significant difference in HDR score between the two groups at epoch 1 (*p* = 0.027) but not at the subsequent timepoints.

**TABLE 4:** Results from the four two‐way repeated measures ANOVAs using episodic memory task scores as the dependent variable and group and epoch as the independent variables

	HIR Task			CIR Task			HDR Task			HRC Task		
Effect	*F*	𝞰^2^	*p*	*F*	𝞰^2^	*p*	*F*	𝞰^2^	*p*	*F*	𝞰^2^	*p*
**Epoch**	6.84	0.13	0.002	0.01	0.00	0.992	11.10	0.19	< 0.001	10.52	0.19	< 0.001
**Group**	3.44	0.07	0.070	1.98	0.04	00.167	4.74	0.09	0.035	2.14	0.04	0.150
**E × G**	0.42	0.01	0.661	1.25	0.03	0.291	0.80	0.02	0.451	1.89	0.04	0.156

E × G represents the interaction term between epoch and group.

Abbreviations: CIR = CANTAB Verbal Memory Immediate Recall, HIR = Hopkins Immediate Recall, HDR = Hopkins Delayed Recall, HRC = Hopkins Recognition Correct.

## 4 Discussion

Contrary to our hypothesis, we did not observe an association between the Aβ group and cognitive decline across all analyzed domains. While the Aβ group x epoch interaction term was associated with reasoning composite score, the cognitive trajectories of the two groups were not dissimilar enough to result in detectable differences in reasoning at the 4‐ or 8‐year follow‐ups, limiting the clinical significance of this effect. The relevance of this finding further diminishes due to the more stringent significance value necessary to account for multiple comparisons. Other notable findings were significant differences in episodic memory composite and HDR task scores between the two groups at epoch 1. This may signify early Aβ‐related episodic memory deterioration, which is in line with the reported vulnerability of episodic memory to AD (Hedden et al. 2013). Despite this, the Aβ group × epoch interaction term was not associated with episodic memory or HDR task score, so this effect is likely weak compared to longitudinal decline. Also, as we expected given the relatively high age of the sample, there was a widespread decline of domain composite and episodic memory task scores over time, excluding the CIR task. In interpreting the null findings, it is important to consider the influence of practice effects. These refer to the amelioration of decline on a given task resulting from prior completion of it, rather than the true slowing of decline on the evaluated cognitive ability (Calamia et al. 2012). If these effects were heterogeneous, they may have obfuscated minor differences in performance between the two groups while sparing more significant longitudinal decline. Additionally, given the relatively small sample, caution should be taken to not overinterpret the null findings, as there remains the possibility of the true smaller effects not detectable in the present study.

A recent study by Mielke et al. (2025) found comparable null associations between plasma Aβ markers and cognitive decline over 8 to 12 years, albeit in a cohort of individuals with risky cardiovascular profiles. These results deserve mention, as their exclusion of individuals without at least 8 years of cognitive data is in line with our methodology, as opposed to other long‐term studies that include individuals with only a few years of cognitive data in mixed‐effects models. Furthermore, in a similar fashion to our observed associations between the Aβ group and episodic memory composite and HDR task scores, De Vito et al. (2025) found an association between Aβ and concurrent cognition but not cognitive decline.

Our results do not support the Aβ‐initiated cognitive decline proposed by the amyloid cascade hypothesis. Given the relatively long observation period, we expected to see broad associations between the Aβ group and cognitive decline, even if we would have ultimately been unable to assert whether such an association was mediated by downstream factors or solely due to Aβ. Thus, our results contribute to the mounting evidence that Aβ is not the sole initiating driver of cognitive decline in AD, though future work modeling additional AD biomarkers and risk factors will permit more robust conclusions, which is also recommended by De Vito et al. (2025).

Despite this conclusion, there are other factors that may have prevented us from detecting broad associations between Aβ group and cognitive decline. Foremost, the dichotomization of Aβ burden is artificial and does not represent the continuous nature of Aβ accumulation. A consequence of this is that participants with Aβ burdens close to the implemented thresholds are more similar than the presence of two distinct groups implies. Though we lowered the upper threshold for the Aβ^−^ group to accommodate this consideration, it is still a point worth noting. There are some limitations to the present study as well. First, given the relatively small sample size, the lack of significance of any given association could have simply been due to a lack of power in our analyses. Future studies should thus confirm these findings using a larger longitudinal sample. Second, though the DLBS cohort is community‐based, the cohort is overrepresented by individuals with higher levels of education, limiting the generalizability of our findings. Third, the DLBS did not record relevant comorbidities, such as hypertension and hyperlipidemia, or family history of dementia or AD. Though blood pressure measurements were completed at the time of cognitive assessment and were not different between the two groups (Table 1), disparities in treatment efficacy and adherence may have confounded this comparison. These two caveats also prevent inferencing the presence of chronic hypertension on the basis of these measurements alone. If participants were unequally distributed in the two groups regarding these comorbidities and family histories, our results may have been influenced. Last, we were unable to control for the presence of an APOE ε4 allele, which has been indicated as a risk factor for AD. This was due to the limited number of Aβ^+^ individuals in the DLBS cohort, a preponderance of whom possess at least one APOE ε4 allele. Still, a greater number of individuals in the Aβ^+^ group (7 heterozygotes and 2 homozygotes) possessed the allele compared to the Aβ^−^ group (3 heterozygotes), which reinforces our findings, as we would reasonably expect that this difference would result in the Aβ^+^ group exhibiting even greater decline over the observation period.

## 5 Conclusion

An association between the Aβ group and cognitive decline was observed in reasoning but not across episodic memory, working memory, and processing speed. However, the present study has limitations, including a relatively small sample size that must be accounted for when considering the null findings to prevent overinterpretation. Our results contribute to the mounting evidence that suggests Aβ is not the sole initiating driver of cognitive decline in AD. Future work should validate our results in larger, similarly controlled samples and model additional AD biomarkers and risk factors to explore different mechanisms for cognitive decline in AD.

## Author Contributions

**Isabella Torres**: data curation, formal analysis, conceptualization, investigation, methodology, validation. **Ekarin Pongpipat**: formal analysis, investigation, visualization. **Evan Smith**: supervision, investigation, writing – review and editing. **Denise Park**: supervision, resources, conceptualization, methodology, investigation, project administration. **Joshua David**: data curation, formal analysis, conceptualization, investigation, writing – original draft, writing – review and editing, methodology, validation, visualization.

## Funding

The authors have nothing to report.

## Ethics Statement

The Dallas Lifespan Brain Study, from which the data used in the study was collected, was conducted in accordance with the University of Texas Southwestern Medical Center (UTSW) and University of Texas at Dallas Institutional Review Boards. All participants provided written informed consent and were debriefed according to human investigations committee guidelines.

## Conflicts of Interest

The authors declare no conflicts of interest

## Data Availability

The data that support the findings of this study are from the Dallas Lifespan Brain Study repository, which is openly available in OpenNeuro at https://openneuro.org/datasets/ds004856/versions/1.0.0.
